# One-Year Functional Decline in COVID-19 and Non-COVID-19 Critically Ill Survivors: A Prospective Study Incorporating a Pre-ICU Status Assessment

**DOI:** 10.3390/healthcare10102023

**Published:** 2022-10-13

**Authors:** Jonathan Cavalleri, Delphine Treguier, Thibaut Deliège, Christine Gurdebeke, Marie Ernst, Bernard Lambermont, Benoit Misset, Anne-Françoise Rousseau

**Affiliations:** 1 Department of Intensive Care, University Hospital of Liège, University of Liège, 4000 Liège, Belgium; 2 Department of Anaesthesiology, University Hospital of Liège, University of Liège, 4000 Liège, Belgium; 3 Intensive Care Unit, Regional Hospital, 4800 Verviers, Belgium; 4 Biostatistics Center (B-STAT), University Hospital of Liège, University of Liège, 4000 Liège, Belgium

**Keywords:** COVID-19, critical illness, post-intensive care syndrome, survivors, survivorship, outcomes, health-related quality of life, activities of daily living, autonomy

## Abstract

We aimed to describe the one-year (1-y) functional status of survivors of COVID-19 critical illness, compared to non-COVID-19 survivors, and compared to their pre-ICU status. Adults who survived a COVID-19 critical illness (COVID group) during the first two waves in 2 hospitals were contacted by phone 1-y after discharge. They were compared to non-COVID-19 ICU survivors. A standardized assessment focused on quality of life (EQ-5D-3L), autonomy for activities of daily living (Barthel Index), and physical activity quantification (IPAQ-SF). Patients rated their 1-y and pre-ICU status. We included 220 survivors (132 COVID and 88 NC). Their age and severity scores were similar. ICU stay was shorter in NC group (3 [3–6] d) than in COVID group (8 [4.2–16.7] d) (*p* = 0.001). Proportions of organ supports were similar in the two groups. At 1-y, a significant reduction in EQ-5D-3L total score, in Barthel Index and in physical activity was observed in both groups, compared to the respective baseline values. Dependency (Barthel < 100) was observed in at least 35% of survivors at 1-y. Independently of the critical illness, HRQoL, autonomy and physical activities at 1-y were still significantly inferior to the pre-ICU values.

## 1. Introduction

Survivors of a critical illness can suffer from mid- and long-term morbidities related to the critical illness itself, the required support, and the environment of the intensive care unit (ICU). These new or worsening disorders negatively affect daily functioning and quality of life in critically ill survivors, have financial consequences for patients and their families, and also have considerable economic implications for society as a whole in terms of, among others, increased healthcare utilization [1].

The COVID-19 acute respiratory distress syndrome (ARDS) has resulted in numerous ICU admissions with prolonged stays. Its management improved after the first wave of the pandemic, and the number of ICU survivors increased significantly [2]. An increasing number of cohort studies reported a significant burden of COVID-19 ARDS among ICU survivors, from ICU discharge up to 12 months after critical illness [3,4,5,6,7,8,9]. A consistent impact on physical and functional capacities has been observed in different long-term studies [10], with variable improvement and recovery compared to earlier assessments [11,12]. However, these data were mostly derived from small cohorts of survivors of the first COVID-19 wave. Less is known about outcomes of survivors of the following waves, in view of the changes in treatment of the COVID-19 ARDS [2]. Furthermore, most of these studies described post-ICU trajectory and lacked precise assessment of pre-ICU status. 

Due to severe hypoxemia and the need for more sophisticated organ support (prone position, neuromuscular blocking agents, extracorporeal membrane oxygenation), the hypothesis of a different pattern of long-term outcomes in ARDS survivors compared to non-ARDS ICU survivors has been raised. However, the available published data are not in favor of specific patterns of post-intensive care syndrome in ARDS survivors compared with other critical illness, at least regarding quality of life [13]. At present, it is not clear if ARDS COVID-19 survivors have a different recovery trajectory compared to non-COVID-19 ICU survivors.

The aim of this bicentric cohort study was to quantify the long-term functional status and activity levels of COVID-19 and non-COVID-19 ICU survivors, compared to their baseline status. Based on our local experience in ICU survivors’ follow-up, we hypothesized that the 1-year functional outcomes would be similar in the two groups. The secondary aim was to analyze the same outcomes separately in COVID-19 patients of the first and second waves of the pandemic. 

## 2. Materials and Methods

### 2.1. Participants

The study was conducted in two public hospitals: a 58-bed academic ICU admitting all types of critically ill patients and a 18-bed non-academic ICU that does not admit patients requiring neuro- or cardiac surgery. These two hospitals are members of the same hospital network and their ICUs collaborate closely. During the COVID-19 pandemic, the two ICUs continued to admit non-COVID critically ill patients, while increasing their capacity up to 71 and 28 ICU beds, respectively. In Belgium, patients benefit from a national health insurance. 

All consecutive critically ill patients surviving an ICU stay for COVID-19 ARDS from 15 March to 30 April 2020 (Wave 1) and from 1 October to 30 November 2021 (Wave 2) were enrolled in the study. There were no a priori exclusion criteria. All patients surviving an ICU stay for any other non-COVID-19 critical illness in March 2021 were also enrolled. Non-COVID-19 patients were excluded in case of prior ICU admission for severe COVID-19 pneumonia during the first months of the pandemic. 

All patients were contacted by phone one year after ICU discharge by the same team. A standardized assessment (defined prior to first inclusion) compared the 1-year functional status to the pre-ICU status (baseline).

The study was reviewed by the Ethics Committee of the University Hospital of Liege (Pr Vincent Seutin, local reference 2021/44). In accordance with Belgian law, as the study did not modify patients’ management and the data were anonymously collected, an oral informed consent was obtained. 

### 2.2. Clinical Variables

Health-related quality of life (HRQoL) was measured using the EQ-5D-3L [14]. This tool comprises two sections: a five-question descriptive component which explores five dimensions: mobility, self-care, usual activities, pain/discomfort and anxiety/depression. Each question has three possible answers, rated from 1 to 3: no, some, or extreme problems. The second section is a visual analogue scale (EQ VAS) of HRQoL. 

Activities of daily living (ADL) were assessed using Barthel Index, a questionnaire measuring functional status and dependency. It consists of 10 subheadings, including feeding, bathing, grooming, dressing, bladder control, bowel control, toilet use, chair–bed transfer, mobility and stair climbing [15]. Scoring ranges from 0–100: a score of 100 is defined as being capable of ADL complete self-care.

Physical activity level was remotely quantified using the French version of the International Physical Activity Questionnaire-Short Form (IPAQ-SF). Patients were asked to report their typical weekly activity types, including vigorous-intensity activities (e.g., heavy lifting, digging, aerobics, or fast bicycling), moderate-intensity activities (e.g., carrying light loads, bicycling at a regular pace, or doubles tennis), walking, and sitting that are undertaken during work, transport, housework, or leisure activities. The total score is the summation of the duration and frequency of walking, moderate-, and vigorous-intensity activities, reported as the “metabolic equivalent of task-min per week (MET-minute/week)” [16]. The IPAQ-SF scores of <600, 600–3000, and >3000 MET-min/week were, respectively, categorized as low, moderate, and high physical activity levels according to the guidelines of the IPAQ (https://sites.google.com/site/theipaq/scoring-protocol, accessed on 30 March 2022).

Finally, patients were questioned about any hospital readmissions, falls and fractures occurring after ICU discharge, about their living condition, and about their return to previous level of activities (employment or leisure activities in unemployed patients).

Demographic data and data related to the ICU stay were collected from the medical charts.

### 2.3. Analysis

In the primary analysis, COVID patients were compared to NC patients. In each group, 1-y outcomes were compared to baseline values.

In a secondary analysis, patients of each group were separated into subgroups: patients who benefited from mechanical ventilation were compared to patients who patients who did not require such respiratory support, and patients who were discharged from hospital directly to inpatient rehabilitation facilities were compared to patients who were discharged to their home. The above-described outcomes were compared between subgroups.

In the COVID group, patients from wave 1 were compared to patients from wave 2.

Finally, in the global cohort, risk factors of functional decline were assessed.

### 2.4. Statistical Analysis

Given the descriptive setting, no a priori sample size was calculated. 

Statistical analysis was performed using Graphpad Prism (version 9.0 for Mac OSX, Graphpad Inc., San Diego, CA, USA) and SAS (version 9.4, SAS institute, Cary, NC, USA). Quantitative variables are expressed as median with first and third quartiles [Q1–Q3], and qualitative variables are described as count and percentage. Comparisons between groups were made using Fisher’s exact test or Chi-square test for categorical variables and using Student t-test or Mann-Whitney test for continuous variables. Paired data were compared using Wilcoxon signed-rank test. Durations were log-transformed. Simple and multiple linear regression models were used to assess the effects of clinical factors on the decline in quality of life, autonomy and physical activity. All variables that had a *p* value lower than a critical level of 0.2 were selected for the stepwise selection in the multivariate model (significant entry and stay levels of 0.15). A value of *p* < 0.05 was considered significant. Missing data were not replaced. 

## 3. Results

### 3.1. Description of the Two Groups

**Figure 1:**
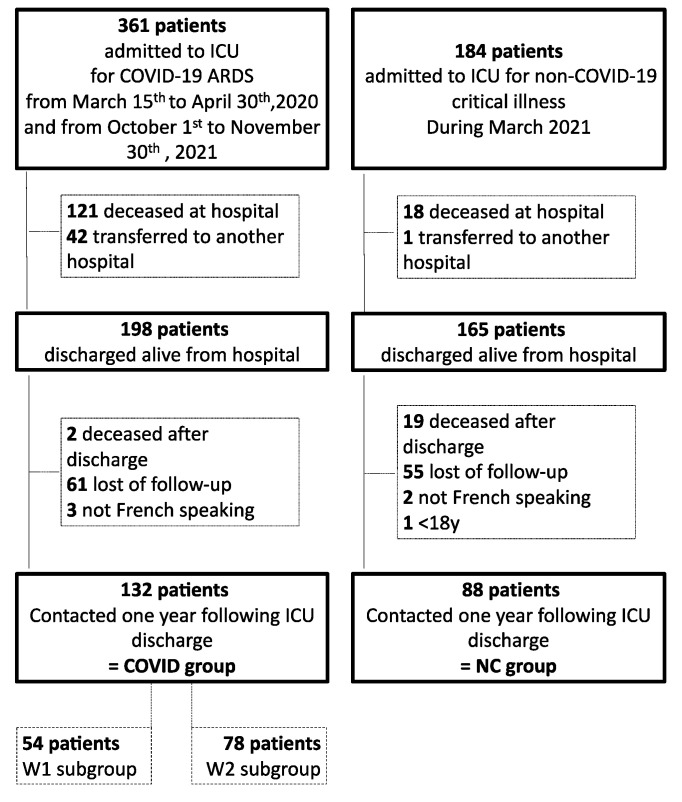
Flow chart (ARDS: acute respiratory distress syndrome; ICU: intensive care unit).

During the study periods, 361 and 184 patients were admitted in ICU, respectively, for COVID-19 ARDS and for non-COVID critical illness. From them, 198 and 165 patients were enrolled in the study and finally, 132 and 88 patients were contacted by phone one year after ICU discharged and were then included in the COVID-19 group (COVID group) and in the non-COVID-19 group (NC group). (Figure 1).

The characteristics of the patients included in the COVID and NC groups are detailed in Table 1. Their age and gender ratio were similar. NC patients had a lower BMI than COVID patients (*p* < 0.001). A higher proportion of NC patients had cardiovascular comorbidities compared to COVID patients (*p* < 0.001), in link with their admission reason. Inversely, the proportion of diabetes was higher in COVID patients compared to NC patients (*p* = 0.002). Severity scores were lower in NC group. COVID patients had a longer ICU length of stay (LOS) (*p* < 0.001) and a longer hospital LOS than NC patients (*p* < 0.001). The proportion of patients who required mechanical ventilation, vasopressor support, and renal replacement therapy was similar in the two groups, but the duration of mechanical ventilation and vasopressor support was longer in the COVID group (*p* < 0.001 and *p* < 0.001, respectively). Prone position was not used in the NC group, whereas it was necessary in about 42.4 % (56/132) of the COVID patients. The proportion of ECMO was similar in the two groups. Tracheostomy was required in a higher proportion in the COVID group than in the NC group (*p* = 0.018).

### 3.2. One-Year Outcomes

**Figure 2:**
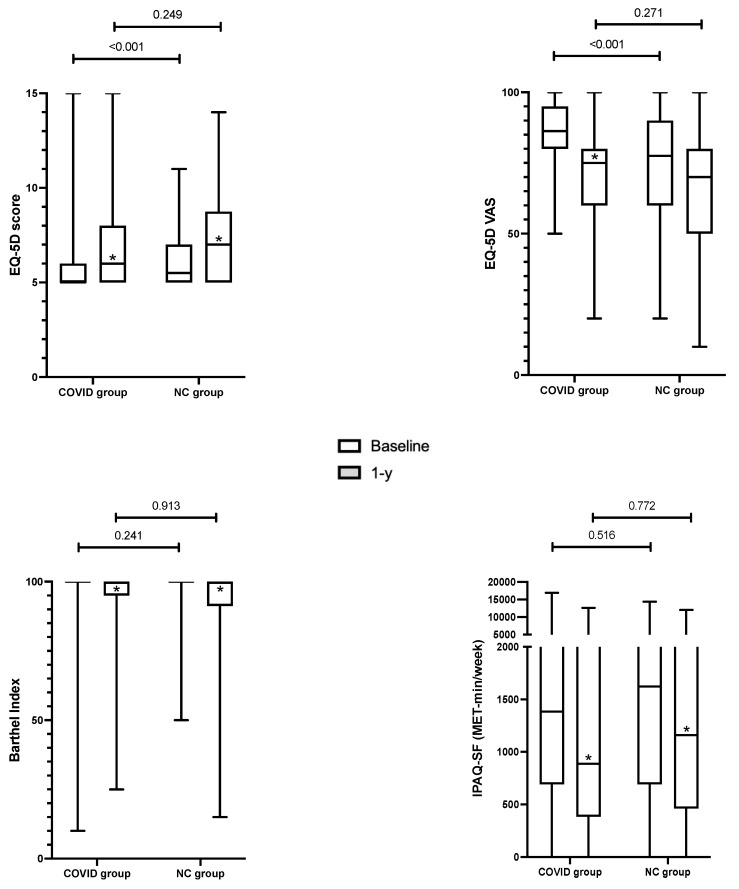
One-year functional assessment (grey boxes) compared to pre-ICU status (baseline, white boxes), in the COVID and NC groups. (*: significant difference between baseline status and one-year status within a group).

Questionnaire results one year after ICU discharge, compared to pre-ICU status, are described in Figure 2. HRQoL was significantly lower at baseline in the NC group compared to the COVID group, both for the EQ-5D score and the EQ-5D EVA (*p* < 0.001 and *p* < 0.001, respectively). The Barthel Index was similar in both groups at baseline (*p* = 0.241). The levels of physical activity were similar at baseline: a low level was observed in 22% of the COVID patients (29/132) and in 17% of the NC patients (15/88), while a high level was observed in 27% of the COVID patients (36/132) and in 32% of the NC patients (*p* = 0.395). The IPAQ-SF score was similar at baseline in the two groups. 

One year after ICU discharge, a significant decrease was observed in the two groups for the four administered questionnaires, excepting for EQ-5D EVA in the NC group. The difference in EQ-5D EVA between baseline and 1-y assessment was −12.9 [−25–0]% and −5.7 [−25–18.9]%, respectively, in the COVID and NC groups (*p* = 0.014). The proportion of patients with incomplete autonomy for ADL (i.e., Barthel Index < 100) increased significantly in both groups: from 12.1% (16/132) at baseline to 36.3% (48/132) at 1-year in the COVID group (*p* < 0.001) and from 18.2% (16/88) to 35.2% (31/88) in the NC group (*p* = 0.016). The difference in physical activity expressed in MET-min/week between baseline and 1-y assessment was −18.1 [−51–0]% and −19.7 [−64.9–0]%, respectively, in the COVID and NC groups (*p* = 0.91).

One year after ICU discharge, 127/132 (96.2%) COVID patients and 85/88 (96.6%) NC patients had returned home (*p* > 0.999). In both groups, nearly half of the patients did not fully return to previous level of activity, either employment for previously active patients or leisure for unemployed or retired patients (Table 2). A quarter of the patients have been readmitted at least once at hospital (all hospital units included) during the year following ICU discharge in the COVID. This proportion was even significantly higher in the NC groups (Table 2). In both groups, cardiovascular disease was the main cause of the first readmission. The occurrence of falls and fractures were similar in the two groups (Table 2).

### 3.3. Subgroup Analysis According to Mechanical Ventilation and Inpatient Rehabilitation

In each group, outcomes of patients who benefited from mechanical ventilation during their ICU stay were compared to those of patients who did not require invasive respiratory support (Table 3). No statistical difference was observed between these two categories of patients in terms of return to previous level of activities, occurrence of hospital readmission, falls and fractures one year after ICU discharge. A functional decline was observed in both MV and non-MV patients in the COVID and NC groups. This decline was non statistically significant for the EQ-5D VAS in the NC group, and for the IPAQ-SF in non-MV NC patients. The functional decline (i.e., the difference between 1-y and baseline results at each score) was of greater importance in MV patients compared to non-MV in the COVID group but was similar between MV and non-MV patients in the NC group.

In each group, 1-year outcomes of patients who were discharged from hospital to a rehabilitation facility were also compared to those of patients who were discharged home in other destinations (Table 4). In these two subgroups, either in the COVID or NC group, a significant loss in HRQoL, autonomy and physical activity was observed one year after discharge compared to baseline. Moreover, in the COVID group, 1-year outcomes were even worse in patients who benefited from an in-patient rehabilitation compared to patients who were discharged home, excepting for physical activity. In the COVID group, patients who benefited from inpatient rehabilitation had a significantly higher ICU LOS (23.5 [10.2–41.5] days) than the other patients (14 [8–20] days) (*p* = 0.20). A higher proportion of these patients were mechanically ventilated during their ICU stay compared to those who were discharged to home (respectively, 36/44 (81.8%) vs. 26/88 (29.5%), *p* < 0.001). On the contrary in the NC group, the proportion of patients under mechanical ventilation during the ICU stay and the ICU LOS were similar, whether they benefited from inpatient rehabilitation or not.

### 3.4. Subgroup Analysis in the COVID Group: Wave 1 vs. Wave 2

Demographics and 1-y assessment in the two subgroups (Wave 1 and Wave 2) are presented in Supplemental Table S1. In W1 subgroup, patients had a longer ICU stay (*p* < 0.001) and more frequently required mechanical ventilation (*p* < 0.001) or vasopressor therapy (*p* < 0.001) than in W2 subgroup. However, the severity scores and the hospital LOS were similar in the two groups. 

The baseline functional status was similar in the two subgroups, excepting the level of physical activities that was slightly higher in the W1 subgroup compared to the W2 subgroup (*p* = 0.049). One year after ICU discharge, the functional evolution was comparable in the two groups: a significant decrease in the four administered questionnaires was observed in both subgroups (Supplemental Figure S1). The proportion of patients with incomplete autonomy, the proportion of patients who fully returned to their previous levels of activities, hospital readmissions, occurrence of falls and fractures were all similar between the two subgroups (Supplemental Table S1).

### 3.5. Risk Factors of Functional Decline

The Supplemental Table S2 depicts the analysis of risk factors for 1-year decline in autonomy for ADL, HR-QoL and physical activity. The independent risk factors for a decrease in the Barthel were CVVH, ICU LOS and hospital LOS. Being retired at ICU admission and hospital LOS were risk factors associated with a decline in HR-QoL expressed as EQ-5D score, and admission failure, ICU LOS and hospital LOS were risk factors for a decline in EQ-5D VAS. Being retired at ICU admission, weight, admission type and ECMO support were risk factors for a decline in physical activity. COVID-19 infection, svereity scores or mechanical ventilation were not found to be risk factor of functional decline one year after ICU discharge.

## 4. Discussion

In this study quantifying long-term autonomy for ADL and physical activity level after critical illness, we observed a similar trajectory during the year following ICU admission for COVID-19 and non-COVID-19 ICU. In the two groups, the burden of the ICU stay was still substantial one year after ICU discharge, expressed by a significant proportion of patients who did not return to their previous level of activities, either professional or leisure. Return to work is a public health issue since at least half of the patients who were employed before ICU admission had not returned to work one year after discharge, whether they suffered from COVID-19 [17] or other critical illness [18]. In the multivariate analysis, COVID-19 was not found to be a risk factor for functional decline one year after ICU discharge. Similarities in post-intensive care syndrome features after an ICU stay, regardless of the primary critical illness, have already been pointed out in some previous studies, 3 (data from our group, under review), 6 [19] and 12 months [20] after discharge.

The present data confirm the findings of a recent observational study in a smaller cohort of COVID-19 patients admitted in ICU during the first and second waves of the pandemic [21]. One year after ICU discharge, half of the survivors reported a lower level of physical exercises compared to pre-ICU status. It is still unclear how physical rehabilitation could enhance and fasten recovery. In the present study, patients who were discharged in a rehabilitation facility after ICU discharge did not have a better post-ICU trajectory than those who were discharged to home. Absence of evidence of rehab benefits is probably due to an important heterogeneity in the studied population, in the rehab program, in the chosen outcomes that may not be relevant enough, and in the measurement tools that may lack standardization. However, physiotherapy is recommended for critically ill patients as early as they are admitted in ICU [22]. It has been recently demonstrated than an early mobilization strategy could positively impact return to ADL one year after discharge [23]. This further emphasizes the well-known role of early mobilization and early physical exercises in post-intensive care syndrome prevention.

From another point of view, the present results can contrast with other optimistic published observations, reporting improvement of physical function one year after ICU discharge, compared with a sooner post-ICU timepoint [11,24,25]. The lack of pre-ICU point of reference could have biased such conclusion. Moreover, it has been suggested that measures of body function or structure (i.e., muscle strength or physical performances) may be less suitable than participation measures (i.e., questionnaires about autonomy or functional performances) to examine patient-centered outcomes, such as HRQoL [26]. In further studies, it would probably be interesting to combine both types of measure to assess more precisely outcomes and effects of interventions.

In this study, the proportion of patients who were readmitted to hospital at least once during the year of follow-up was higher in the non-COVID-19 group, reaching 47%. This agrees with the results of a recent meta-analysis showing that up to half of ICU survivors experienced acute hospital readmission in the year following discharge [27]. In addition, HRQoL seemed to be less impacted in non-COVID-19 survivors, knowing that their pre-ICU HRQoL was lower than COVID-19 critically ill patients. These observations probably reflect the more complex pre-ICU trajectories in non-COVID-19 patients, who are finally admitted in ICU with end-stage organ dysfunctions and a severe comorbid status.

Despite a drastic change in clinical management of COVID-19 ARDS after the first wave including systematic dexamethasone administration [2], and a subsequent reduction in ICU LOS and needs for mechanical ventilation, functional outcomes and post-ICU trajectories were not affected by a wave-effect in the present cohort. This may be explained by the multifactorial nature of post-intensive care syndrome. Occurrence of post-ICU disabilities is not only the consequence of ICU factors, but also involves patient factors and systemic factors that are not necessarily closely linked to the primary critical illness, such as age, past medical history, pre-ICU functional status or social support and access to rehabilitation services. ICU factors are risk factors and not causal factors in a mathematical relationship. In particular, in the present study, an absence of mechanical ventilation did not seem to have a protective effect on functional decline in neither group.

Using a suitable method and validated questionnaires, this study extends the knowledge regarding post-ICU functional outcomes and the burden of an ICU stay after considering the baseline status of the survivors. However, some limitations need to be acknowledged. First, there was no sample size calculation and samples were relatively small. Second, outcomes were assessed by phone calls and not during a face-to-face consultation, limiting the use of objective measurements or biological markers. On the contrary, a remote assessment strategy may have limited the number of participants lost to follow-up related to difficulties to attend an in-person consultation and thus may have increased the response rate. Moreover, such a remote assessment can only be focused on patient-reported outcomes that best capture information about patients’ experiences of their health condition, differing from other objective measures [28]. Third, the control group was not limited to non-COVID-19 ARDS but included all causes of non-COVID-19 critical illness. The NC group was not a priori matched by age and sex with COVID group. However, the two groups were similar in term of age, sex and severity scores. This unmatched case-control study reflects the daily life of a post-ICU follow-up clinic and confirms that both the COVID and NC survivors could have similar requirements in terms of post-ICU support. Fourth, confounding factors that may have impacted quality of life or autonomy were not considered. This could lead to a misinterpretation of the role of critical illness and ICU stay in the recovery trajectory. Fifth, some other data potentially modifying the post-ICU trajectory were not considered, such as mild COVID-19 infection in the NC group or characteristics of any home care delivery. Finally, baseline (pre-ICU) status was retrospectively assessed, introducing a potential memory bias. Measurement of pre-ICU physical or functional status is always difficult to obtain in real time for most of the admitted patients. However, the validity of retrospective measurement of functional outcomes (especially HRQoL) to estimate pre-morbid health status has already been demonstrated in previous studies [29].

## 5. Conclusions

One year after discharge, the burden of an ICU stay on HRQoL, autonomy and physical activities was still substantial, especially when compared to the pre-ICU condition. A COVID-19 ARDS was not found to be a specific risk factor for functional decline. The present observations should encourage ICU teams to enroll survivors into an individualized follow-up. Further research should aim to specify how follow-up and rehabilitation could be targeted to unique patient needs, and how such strategy could improve and fasten recovery.

## Figures and Tables

**Table 1:** Demographics in the COVID and NC groups.

Data		COVID Group *n* = 132	NC Group *n* = 88	*p* Value
Age, y		64 [54–70]	66 [57–72.5]	0.13
Male, *n* (%)		85 (64.4)	63 (71.6)	0.20
Weight, kg		88.4 [78.5–100]	76 [68–90]	<0.001
BMI, kg/m^2^		30.3 [26.5–34]	26.7 [23.1–30.3]	<0.001
Comorbidities, *n* (%)	Cardiovascular ^a^	49 (37.1)	67 (76.1)	<0.001
	Hypertension ^b^	82 (62.1)	62 (70.5)	0.20
	Respiratory	28 (21.2)	19 (21.6)	0.95
	Chronic kidney disease	19 (14.4)	10 (11.4)	0.52
	Diabetes	62 (47)	23 (26.1)	*0.002*
	Immunosuppression	6 (4.5)	2 (2.3)	0.48
Retired before ICU admission, *n* (%)		70 (53)	54 (61.4)	0.22
Admission type, *n* (%)	Medical	132 (100)	33 (37.5)	
	Surgical	-	55 (62.5)	
Admission failure, *n* (%)	Cardiovascular	-	48 (54.5)	
	Pulmonary	132 (100)	10 (11.4)	
	Neurologic	-	10 (11.4)	
	Other	-	20 (22.7)	
SOFA at admission		4 [3–6]	3 [1–5]	0.011
SAPS II		34 [26–46]	29 [23–36]	0.024
Mechanical ventilation, *n* (%)		62 (46.9)	47 (53.4)	0.35
Duration of mechanical ventilation, d		13 [8–23]	1 [1–2]	*<0.001*
Neuromuscular blocking agent, *n*(%)		34 (25.8)	12 (13.6)	*0.030*
Duration of NMBA, d		3 [2–4]	1 [1–3]	*0.021*
Tracheostomy, *n* (%)		15 (11.3)	2 (2.3)	*0.013*
Vasopressor support, *n* (%)		46 (34.8)	33 (37.5)	0.69
Duration of norepinephrine administration, d		3 [2–9]	1 [1–2]	*<0.001*
Renal replacement therapy, *n* (%)		10 (7.6)	2 (2.2)	0.13
Duration of renal replacement therapy, d		19 [18–35]	3 and 9	
Extracorporeal membrane oxygenation, *n* (%)		0	1 (1.1)	0.40
Duration of ECMO, d		-	3	
ICU LOS, d		8.5 [4.5–20]	2 [2–4]	*<0.001*
Hospital LOS, d		22 [12–42]	11 [8–24]	*<0.001*
Destination at hospital discharge, *n* (%)	Home	83 (62.9)	66 (75)	0.071
	Rehabilitation facility	44 (33.3)	17 (19.3)	
	Other	5 (3.8)	5 (5.7)	

Data are presented as count and percentage or as median with first and third quartiles [Q1–Q3]. BMI: body mass index; ECMO: extracorporeal membrane oxygenation; ICU: intensive care unit, LOS: length of stay; SAPS II: Simplified Acute Physiology Score; SOFA: Sequential Organ Failure Assessment. ^a^ Ischemic heart disease, valvular disease, cardiomyopathies, and chronic heart disease. ^b^ Asthma, chronic obstructive pulmonary disease and interstitial lung diseases.

**Table 2:** One-year outcomes in the COVID and NC groups.

Data		COVID Group *n* = 132	NC Group *n* = 88	*p* Value
Return to previous level of activities, *n* (%)		76 (57.5)	45 (51.1)	0.407
At least one hospital readmission, *n* (%)		34 (25.7)	41 (46.6)	*0.002*
Reason for the first readmission, *n* (%)	Cardio-vascular	9 (26.5)	11 (26.9)	
	Pulmonary	3 (8.8)	1 (2.4)	
	Neurologic	1 (2.9)	8 (19.5)	
	Trauma	4 (11.8)	1 (2.4)	
	Scheduled surgery	7 (20.6)	3 (7.3)	
	Sepsis	7 (20.6)	6 (14.6)	
	Other	3 (8.8)	11 (26.9)	
At least one fall, *n* (%)		16 (12.1)	13 (14.8)	0.685
At least one fracture, *n* (%)		3 (2.3)	5 (5.7)	0.271

Data are presented as counts and percentages.

**Table 3:** One-year outcomes in the COVID and NC groups, whether patients required mechanical ventilation or not during their ICU stay.

Data		COVID Group *n* = 132		NC Group *n* = 88	
		MV (*n* = 62)	Non-MV (*n* = 70)	MV (*n* = 47)	Non-MV (*n* = 41)
Return to previous level of activities, *n* (%)		33 (53.2)	42 (60)	25 (53.2)	20 (48.8)
		*p* = 0.483		*p* = 0.831	
At least one hospital readmission, *n* (%)		18 (29)	14 (20)	26 (55.3)	15 (36.6)
		*p* = 0.309		*p* = 0.091	
At least one fall, *n* (%)		11 (17.7)	5 (7.1)	5 (10.6)	8 (19.5)
		*p* = 0.107		*p* = 0.38	
At least one fracture, *n* (%)		3 (4.8)	0	2 (4.3)	3 (7.3)
		*p* = 0.101		*p* = 0.661	
EQ-5D score	baseline	5 [5–6]	5 [5–5]	6 [5–7]	5 [5–7]
	1-y	7 [6–9] *	6 [5–7] *	7 [6–9] *	7 [5–8] *
EQ-5D VAS	baseline	90 [80–100]	85 [73.8–90]	75 [60–90]	80 [60–85]
	1-y	72.5 [60–80] *	75 [60–89.2] *	75 [50–80]	70 [60–82.5]
Barthel Index	baseline	100 [100–100]	100 [100–100]	100 [100–100]	100 [100–100]
	1-y	97.5 [90–100] *	100 [98.7–100] *	100 [90–100] *	100 [95–100] *
IPAQ-SF (MET-min/week)	baseline	1248 [678.8–3959]	1413 [689.3–2410]	1512 [693–3115]	1732 [693–3333]
	1-y	784.5 [333.8–2946] *	1093 [427.5–1985] *	900 [462–2079] *	1386 [288.5–2948]

Data are presented as count and percentage or as median with first and third quartiles [Q1–Q3]. MV: mechanical ventilation. *: significant difference between baseline value and 1-y value in a same subgroup.

**Table 4:** One-year outcomes in the COVID and NC groups, whether patients required whether they benefited from rehabilitation after ICU discharge or not.

Data		COVID Group *n* = 132		NC Group *n* = 88	
		Rehab (*n* = 44)	No Rehab (*n* = 88)	Rehab (*n* = 17)	No Rehab (*n* = 71)
Return to previous level of activities, *n* (%)		19 (43.2)	57 (64.8)	7 (41.2)	38 (53.5)
		*p = 0.025*		*p* = 0.424	
At least one hospital readmission, *n* (%)		15 (34.1)	19 (21.6)	8 (47.1)	33 (46.5)
		*p* = 0.142		*p* > 0.999	
At least one fall, *n* (%)		9 (20.4)	7 (7.9)	4 (23.5)	9 (12.7)
		*p = 0.049*		*p* = 0.267	
At least one fracture, *n* (%)		2 (4.5)	1 (1.1)	1 (5.9)	4 (5.6)
		*p* = 0.258		*p* > 0.999	
EQ-5D score	baseline	5 [5–5]	5 [5–6]	6 [5–7]	5 [5–7]
	1-y	7.5 [6–9] *	6 [5–7.8] *,‡	8 [6–10] *	7 [5–8] *
EQ-5D VAS	baseline	90 [80–100]	85 [75.2–93.7]	80 [60–90]	75 [60–90]
	1-y	70 [52.5–80] *	75 [65–85] *,‡	70 [45–75] *	70 [60–80]
Barthel Index	baseline	100 [100–100]	100 [100–100]	100 [100–100]	100 [100–100]
	1-y	95 [85–100] *	100 [95–100] *,‡	100 [77.5–100] *	100 [95–100] *
IPAQ-SF (MET-min/week)	baseline	1494 [743.6–3968]	1293 [604.5–2611]	960 [351–2038]	1820 [693–3192]
	1-y	842 [393.5–3026] *	936 [376.5–1995] *	462 [16.5–1143] *	1386 [480–2555] *,‡

Data are presented as count and percentage or as median with first and third quartiles [Q1–Q3]. MV: mechanical ventilation. *: significant difference between baseline value and 1-y value in a same subgroup. ‡: significant difference between 1-y values in the two subgroups.

## Data Availability

The datasets used and/or analyzed during the current study are available from the corresponding author on reasonable request.

## Supplementary Materials

The following supporting information can be downloaded at: https://www.mdpi.com/article/10.3390/healthcare10102023/s1, Table S1: One-year outcomes in the COVID and NC groups; Table S2: Demographics and 1-year outcomes in Wave 1 and Wave 2 subgroups in the COVID group; Figure S1: One-year functional assessment compared to pre-ICU status, in the Wave 1 and Wave 2 subgroups in the COVID group.
