# Supplementary material for: One-Year Functional Decline in COVID-19 and Non-COVID-19 Critically Ill Survivors: A Prospective Study Incorporating a Pre-ICU Status Assessment
Source: Healthcare (Basel). 2022 Oct 13;10(10):2023. doi: 10.3390/healthcare10102023 (PMC9602164; doi:10.3390/healthcare10102023)

One-year functional decline in COVID-19 and non-COVID-19 critically ill survivors: a prospective study incorporating a pre-ICU status assessment.

Supplemental materials

Supplemental Table S1: Demographics and 1-year outcomes in Wave 1 and Wave 2 subgroups in the COVID group

Data are presented as count and percentage or as median with first and third quartiles [Q1-Q3].

BMI: body mass index; ICU: intensive care unit, LOS: length of stay; SAPS II: Simplified Acute Physiology Score; SOFA: Sequential Organ Failure Assessment

Data	Wave 1 subgroup n=54	Wave 2 subgroup n=78	p value
Demographics			
Age, y	64 [50-70]	63.5 [54.7-71]	0.309
Male, n (%)	35 (64.8)	50 (64.1)	>0.999
Weight, kg	91 [80-104]	86 [77.2-98.8]	*0.034*
BMI, kg/m^2	30.9 [27.5-35]	30.1 [26.1-33.8]	0.103
Retired before ICU admission, n (%)	28 (51.8)	42 (53.8)	0.864
SOFA at admission	4 [3-6]	4 [3-5]	0.371
SAPS II	34.5 [26-43]	32.5 [26-50]	0.811
Mechanical ventilation, n (%)	38 (70.3)	25 (32.1)	*<0.001*
Duration of mechanical ventilation, d	16 [10-23]	9 [4-28]	0.546
Neuromuscular blocking agent, n(%)	12 (22.2)	22 (28.2)	0.545
Duration of NMBA, d	3 [3-3.5]	2.5 [1-5]	0.283
Tracheostomy, n (%)	9 (16.7)	6 (7.7)	0.162
Vasopressor support, n (%)	28 (51.9)	18 (23.1)	*<0.001*
Duration of norepinephrine administration, d	3 [2-9]	1.5 [1-9.2]	0.09
Renal replacement therapy, n (%)	6 (11.1)	4 (5.1)	0.316
Duration of renal replacement therapy, d	18 [17-19]	37 [35-39]	0.2
Extracorporeal membrane oxygenation, n (%)	0	0	

ICU LOS, d	15.5 [6-27]	6 [4-11]	*<0.001*
Hospital LOS, d	27 [14-46]	17.5 [12-36.2]	0.121
1-year outcomes			
Barthel Index < 100, n (%)	22 (40.7)	26 (33.3)	0.4623
Return to previous level of activities, n (%)	34 (63)	42 (53.8)	0.371
At least one hospital readmission, n (%)	14 (25.9)	20 (25.6)	>0.999
At least one fall, n (%)	4 (7.4)	12 (15.4)	0.188
At least one fracture, n (%)	1 (1.9)	2 (2.6)	>0.999

Supplemental Table S2: Risk factors for functional decline in the global cohort.

BMI: body mass index; CKD: chronic kidney disease; ECMO: extracorporeal membrane oxygenation; ICU: intensive care unit, LOS: length of stay; SAPS II: Simplified Acute Physiology Score; SOFA: Sequential Organ Failure Assessment

Coeff: regression coefficient; SE: standard error

R^2 measures the strength of the relationship between the linear model and the dependent variable (0-100%)

		Univariate				Multivariate				
**Variable**	**Explicative factor**	**N**	**Coeff**	**SE**	**p-value**	**N**	**R^2 (%)**	**Coeff**	**SE**	**p-value**
Barthel index						216	20.0			
	Age, y	220	-0.21	0.07	0.0037			-0.12	0.07	0.072
	Sex, Male	220	2.19	1.92	0.25					
	Weight, kg	217	-0.03	0.05	0.50					
	BMI, kg/m^2	216	-0.15	0.15	0.32					
	Retired before ICU admission	220	-2.69	1.80	0.14					
	Comorbidities	220	-2.24	2.65	0.40					
	CKD (yes vs no)	220	-0.16	1.85	0.93					
	Diabetes (yes vs no)	220	-3.55	1.88	0.059					
	Hypertension (yes vs no)	220	0.62	1.80	0.73					
	Cardiovascular (yes vs no)	220	-3.02	2.19	0.17					
	Respiratory (yes vs no)	219	-3.16	4.81	0.51					
	Immunosuppression (yes vs no)	220	-1.05	0.73	0.15					
	Total number of these comorbidities (0 to 6)									
	Admission type (surgical vs medical)	220	1.67	2.07	0.42					
	Admission failure	220			0.93					
	Cardiovascular (vs pulmonary)		0.27	2.24						
	Neurologic (vs pulmonary)		-2.56	4.38						
	Other (vs pulmonary)		0.69	3.20						
	SOFA at admission	147	-0.53	0.40	0.19					
	SAPS II	132	-0.16	0.09	0.069					
	COVID-19 (yes vs no)	220	0.95	1.83	0.61					
	Mechanical ventilation (yes vs no)	220	-4.94	1.77	0.0056					
	Neuromuscular blocking agent (yes vs no)	220	-5.25	2.18	0.017					
	Tracheostomy (yes vs no)	220	-4.76	3.35	0.16					
	Vasopressor support (yes vs no)	220	-5.13	1.84	0.0058					
	Renal replacement therapy (yes vs no)	220	-12.94	3.86	0.0010			-12.86	4.32	0.0032
	ECMO (yes vs no)	220	4.45	13.37	0.74					
	ICU LOS, d (log)	220	-1.31	0.83	0.12			2.92	1.37	0.034
	Hospital LOS, d (log)	216	-4.47	0.98	<0.0001			-4.04	1.59	0.012
	Home at hospital discharge (yes vs no)	220	11.45	3.28	0.0006			6.23	3.32	0.062
	Rehabilitation (yes vs no)	220	-8.50	1.93	<0.0001			-4.20	2.36	0.077
EQ-5D score						216	19.3			
	Age, y	220	0.00	0.01	0.84					
	Sex, Male	220	0.03	0.28	0.92					
	Weight, kg	217	0.01	0.01	0.061					
	BMI, kg/m^2	216	0.03	0.02	0.20					
	Retired before ICU admission	220	-0.48	0.26	0.066			-0.59	0.25	0.019
	Comorbidities									
	CKD (yes vs no)	220	0.00	0.38	0.99					
	Diabetes (yes vs no)	220	0.37	0.26	0.16					
	Hypertension (yes vs no)	220	0.02	0.27	0.95					
	Cardiovascular (yes vs no)	220	-0.41	0.26	0.11					
	Respiratory (yes vs no)	220	0.49	0.31	0.12			0.46	0.29	0.12
	Immunosuppression (yes vs no)	219	-0.46	0.69	0.51					

Variable	Explicative factor	N	Univariate Coeff	SE	p-value	Multivariate N	R^2 (%)	Coeff	SE	p-value
	Total number of these comorbidities (0 to 6)	220	0.04	0.11	0.72					
	Admission type (surgical vs medical)	220	-0.64	0.30	0.032					
	Admission failure	220			0.25					
	Cardiovascular (vs pulmonary)		-0.54	0.32						
	Neurologic (vs pulmonary)		0.41	0.62						
	Other (vs pulmonary)		0.21	0.46						
	SOFA at admission	147	0.07	0.06	0.31					
	SAPS II	132	0.03	0.01	0.051					
	COVID-19 (yes vs no)	220	0.16	0.26	0.54					
	Mechanical ventilation (yes vs no)	220	0.60	0.26	0.020					
	Neuromuscular blocking agent (yes vs no)	220	1.25	0.31	<0.0001			0.67	0.35	0.055
	Tracheostomy (yes vs no)	220	0.62	0.48	0.20			-0.77	0.52	0.14
	Vasopressor support (yes vs no)	220	0.75	0.26	0.0047					
	Renal replacement therapy (yes vs no)	220	1.52	0.56	0.0072					
	ECMO (yes vs no)	220	4.71	1.90	0.014			2.79	1.84	0.13
	ICU LOS, d (log)	220	0.48	0.13	0.0003					
	Hospital LOS, d (log)	216	0.72	0.14	<0.0001			0.64	0.16	0.0001
	Home at hospital discharge (yes vs no)	220	-1.06	0.48	0.027			-0.76	0.48	0.11
	Rehab (yes vs no)	220	1.22	0.28	<0.0001					
EQ-5D VAS						216	18.6			
	Age, y	220	-0.01	0.12	0.92					
	Sex, Male	220	0.82	3.11	0.79					
	Weight, kg	217	0.00	0.08	0.95					
	BMI, kg/m^2	216	0.08	0.24	0.74					
	Retired before ICU admission	220	4.09	2.92	0.16			4.33	2.81	0.12
	Comorbidities									
	CKD (yes vs no)	220	0.30	4.30	0.94					
	Diabetes (yes vs no)	220	-7.43	2.95	0.012			-4.41	2.79	0.12
	Hypertension (yes vs no)	220	3.83	3.05	0.21					
	Cardiovascular (yes vs no)	220	8.73	2.86	0.0025					
	Respiratory (yes vs no)	220	-1.38	3.55	0.70					
	Immunosuppression (yes vs no)	219	0.14	7.78	0.99					
	Total number of these comorbidities (0 to 6)	220	0.73	1.19	0.54					
	Admission type (surgical vs medical)	220	9.80	3.30	0.0033					
	Admission failure	220			0.0010					0.020
	Cardiovascular (vs pulmonary)		13.42	3.49				9.50	5.46	
	Neurologic (vs pulmonary)		-6.18	6.84				-0.62	4.74	
	Other (vs pulmonary)		2.82	4.99				-8.10	7.98	
	SOFA at admission	147	-0.34	0.70	0.62					
	SAPS II	132	-0.13	0.15	0.39					
	COVID-19 (yes vs no)	220	-8.65	2.91	0.0033					
	Mechanical ventilation (yes vs no)	220	-4.91	2.89	0.091					
	Neuromuscular blocking agent (yes vs no)	220	-11.49	3.49	0.0012					
	Tracheostomy (yes vs no)	220	-11.88	5.39	0.029					
	Vasopressor support (yes vs no)	220	-4.95	3.02	0.10					
	Renal replacement therapy (yes vs no)	220	-16.75	6.31	0.0085					
	ECMO (yes vs no)	220	-35.24	21.51	0.10					
	ICU LOS, d (log)	220	-4.51	1.47	0.0025					
	Hospital LOS, d (log)	216	-7.27	1.57	<0.0001			-5.07	1.66	0.0025
	Home at hospital discharge (yes vs no)	220	13.54	5.38	0.012			15.86	5.38	0.0036
	Rehab (yes vs no)	220	-12.00	3.15	0.0002					
IPAQ-SF (MET-min/week)						217	12.1			
	Age, y	220	20.1	10.6	0.06					
	Sex, Male	220	-337.5	284.2	0.24					
	Weight, kg	217	-14.1	7.1	0.049			-13.9	7.0	0.049
	BMI, kg/m^2	216	-12.3	21.7	0.57					
	Retired before ICU admission	220	767.3	263.7	0.0040			679.2	262.2	0.010
	Comorbidities									
	CKD (yes vs no)	220	878.9	389.4	0.025			664.8	381.6	0.083
	Diabetes (yes vs no)	220	-42.4	273.7	0.88					
	Hypertension (yes vs no)	220	443.5	278.7	0.11					
	Cardiovascular (yes vs no)	220	52.4	266.9	0.84					
	Respiratory (yes vs no)	220	341.9	324.3	0.29					
	Immunosuppression (yes vs no)	219	401.2	713.0	0.57					

Variable	Explicative factor	Univariate N	Coeff	SE	p-value	Multivariate N	R² (%)	Coeff	SE	p-value
	Total number of these comorbidities (0 to 6)	220	184.4	108.4	0.09					
	Admission type (surgical vs medical)	220	-423.8	306.4	0.17			-607.0	306.7	0.049
	Admission failure	220			0.15					
	Cardiovascular (vs pulmonary)		-221.9	327.5						
	Neurologic (vs pulmonary)		-1175.9	641.8						
	Other (vs pulmonary)		504.6	468.5						
	SOFA at admission	147	37.5	62.2	0.55					
	SAPS II	132	2.1	12.6	0.87					
	COVID-19 (yes vs no)	220	195.4	271.7	0.47					
	Mechanical ventilation (yes vs no)	220	-185.7	266.3	0.49					
	Neuromuscular blocking agent (yes vs no)	220	-14.0	327.7	0.97					
	Tracheostomy (yes vs no)	220	-54.6	499.1	0.91					
	Vasopressor support (yes vs no)	220	-5.3	277.8	0.98					
	Renal replacement therapy (yes vs no)	220	166.5	586.8	0.78					
	ECMO (yes vs no)	220	-6160.8	1936.8	0.0017			-5529.9	1899.4	0.0040
	ICU LOS, d (log)	220	-50.1	137.7	0.72					
	Hospital LOS, d (log)	216	62.1	153.5	0.69					
	Home at hospital discharge (yes vs no)	220	387.0	498.4	0.44					
	Rehab (yes vs no)	220	-271.4	297.2	0.36					

Supplemental Figure S1. One-year functional assessment (grey boxes) compared to pre-ICU status (baseline, white boxes), in the Wave 1 and Wave 2 subgroups in the COVID group.

**: significant difference between baseline status and 1-y status within a group*

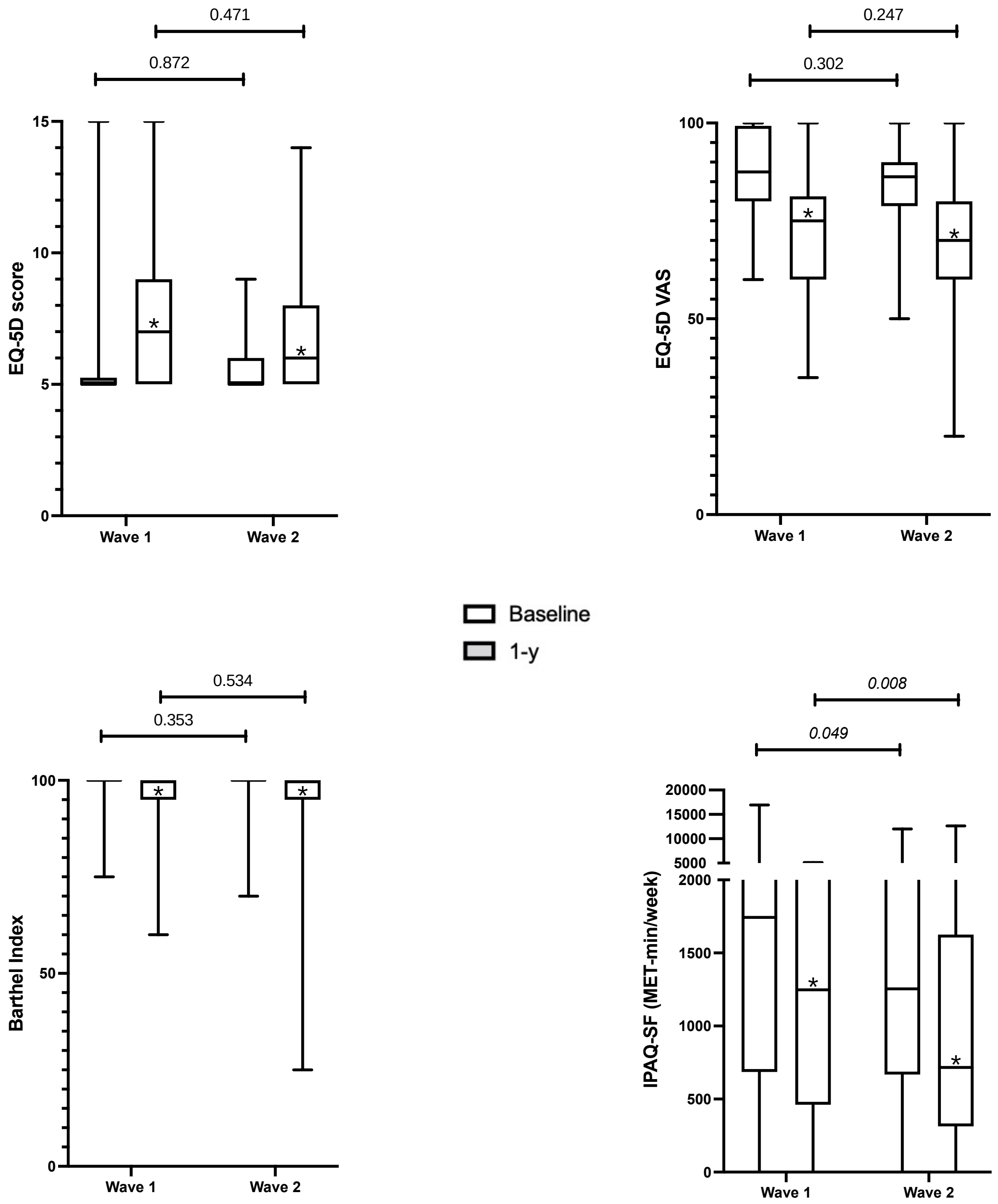

Supplement: Supplementary file 1 [file healthcare-10-02023-s001.zip › healthcare-1929831-supplementary.pdf]
